# Insights into the Conformation of the Membrane Proximal Regions Critical to the Trimerization of the HIV-1 gp41 Ectodomain Bound to Dodecyl Phosphocholine Micelles

**DOI:** 10.1371/journal.pone.0160597

**Published:** 2016-08-11

**Authors:** John M. Louis, James L. Baber, Rodolfo Ghirlando, Annie Aniana, Ad Bax, Julien Roche

**Affiliations:** 1 Laboratory of Chemical Physics, National Institute of Diabetes and Digestive and Kidney Diseases, National Institutes of Health, Bethesda, Maryland, United States of America; 2 Laboratory of Molecular Biology, National Institute of Diabetes and Digestive and Kidney Diseases, National Institutes of Health, Bethesda, Maryland, United States of America; 3 Roy J. Carver Department of Biochemistry, Biophysics and Molecular Biology, Iowa State University, Ames, Iowa, United States of America; German Primate Center, GERMANY

## Abstract

The transitioning of the ectodomain of gp41 from a pre-hairpin to a six-helix bundle conformation is a crucial aspect of virus-cell fusion. To gain insight into the intermediary steps of the fusion process we have studied the pH and dodecyl phosphocholine (DPC) micelle dependent trimer association of gp41 by systematic deletion analysis of an optimized construct termed 17–172 (residues 528 to 683 of Env) that spans the fusion peptide proximal region (FPPR) to the membrane proximal external region (MPER) of gp41, by sedimentation velocity and double electron-electron resonance (DEER) EPR spectroscopy. Trimerization at pH 7 requires the presence of both the FPPR and MPER regions. However, at pH 4, the protein completely dissociates to monomers. DEER measurements reveal a partial fraying of the C-terminal MPER residues in the 17–172 trimer while the other regions, including the FPPR, remain compact. In accordance, truncating nine C-terminal MPER residues (675–683) in the 17–172 construct does not shift the trimer-monomer equilibrium significantly. Thus, in the context of the gp41 ectodomain spanning residues 17–172, trimerization is clearly dependent on FPPR and MPER regions even when the terminal residues of MPER unravel. The antibody Z13e1, which spans both the 2F5 and 4E10 epitopes in MPER, binds to 17–172 with a *K*_d_ of 1 ± 0.12 μM. Accordingly, individual antibodies 2F5 and 4E10 also recognize the 17–172 trimer/DPC complex. We propose that binding of the C-terminal residues of MPER to the surface of the DPC micelles models a correct positioning of the trimeric transmembrane domain anchored in the viral membrane.

## Introduction

**Fig 1:**
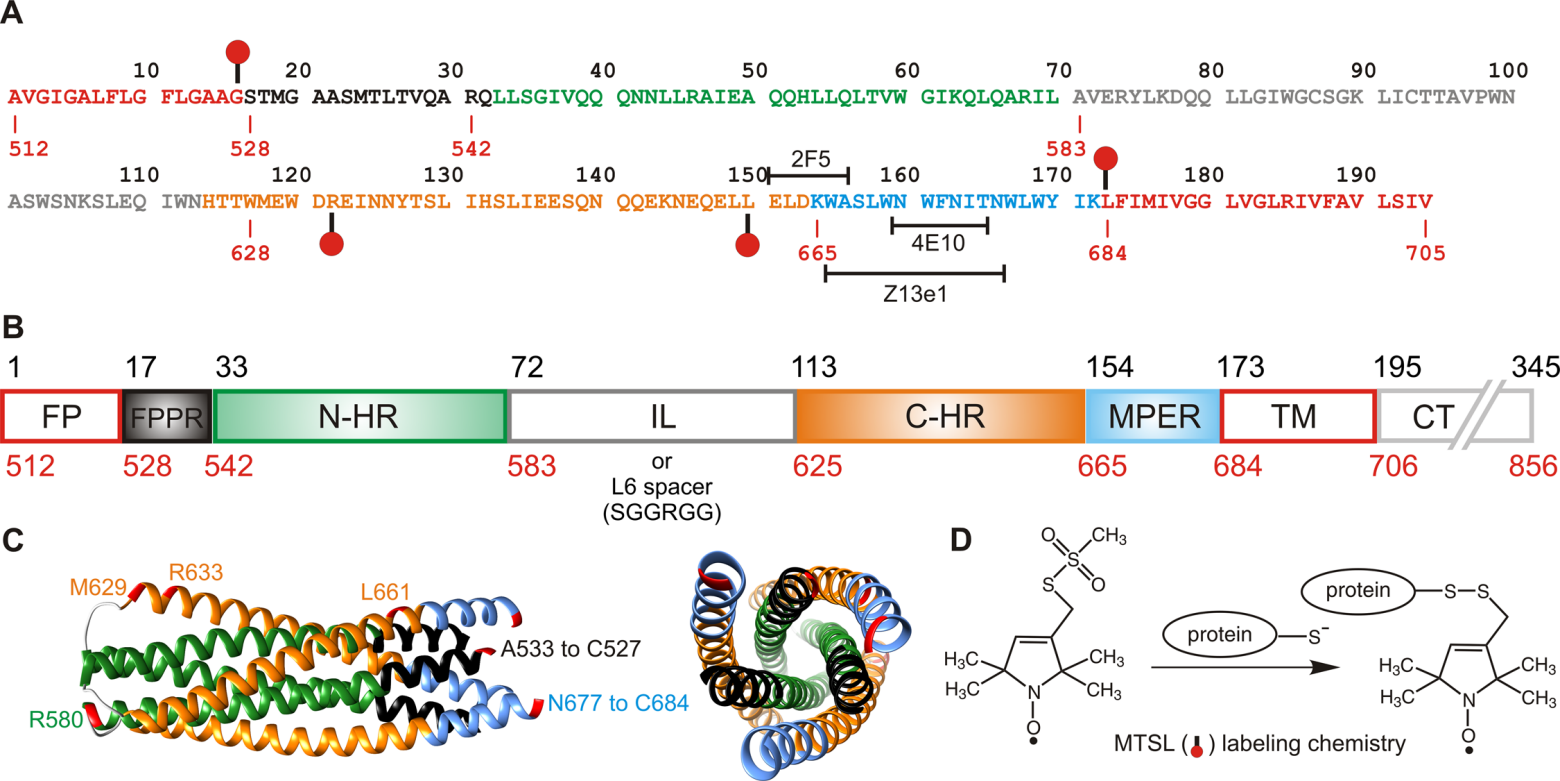
Amino acid sequence, domain organization and structure representations of HIV-1 gp41. (A) Numbering of residues corresponds to their positions in the HIV-1 Env sequence. Gp41 spans residues 512 to 856 (in red) of gp160. (B) Abbreviations are as follows: FP, fusion peptide; FPPR, fusion peptide proximal region; N-HR, N-heptad repeat; IL, immune-dominant linker; C-HR, C-heptad repeat; MPER, membrane proximal external region; TM, transmembrane region; and CT, intraviral C-terminal domain, respectively. For ease of designating the various constructs used in this study, gp41 is renumbered from 1–345 (in black). The longest sequence used in this study spans residues 17 to 194 corresponding to Env numbering 528 to 705. Except for 35-144^IL^, all constructs have their IL region (residues 69 through 116) replaced with the L6 spacer (SGGRGG) (S1 Fig). They are designated based on the amino acid sequence they encompass. When not substituted with the L6 spacer, the construct is designated with an IL (in superscript) following the designation. The various constructs used are shown in S1 Fig (C). The 6HB spanning A533 to R580 (FPPR/N-HR) and M629-N677 (C-HR/MPER) is modeled from pdb entries 1SZT [14] and 2X7R [17]. Terminal residues are indicated in red on the ribbon. Positions substituted with cysteines for MTSL-labeling (chemistry shown in D) are R633, L661, G527 and L684. These positions are indicated also in red on the ribbon (in C) and the sequence with a ball and stick (in A). The epitopes recognized by the antibodies 2F5, 4E10 and Z13e1 are shown in (A).

The fusion of the human immunodeficiency virus (HIV) with the target cell membrane is mediated by the viral glycoprotein gp120 and transmembrane gp41 complex processed from the full length envelope precursor gp160 [1,2]. The domain organization of gp41 is shown in Fig 1. Each subunit of the gp41 trimer interacts with a gp120 subunit forming a non-covalent trimer of heterodimers [3]. Interaction of gp120 with CD4 and the chemokine coreceptor on the target cell initiates a series of conformational changes yielding an extended pre-hairpin gp41 conformation [4,5]. Such complexes, thought to represent an “activated or open” state, have recently been visualized by single molecule FRET, cryo-electron microscopy and crystallography [6,7,8]. In this prefusion state the N-terminal fusion peptide (FP) is expected to contact the host cell membrane, while its transmembrane region (TM) forms a trimer anchored to the viral membrane [9]. Transitioning from a pre-hairpin state to a six-helix bundle through interactions of the immune-dominant linker (IL) and the N- and C-terminal heptad repeats (N-HR and C-HR, respectively) is crucial for initial fusion events that ultimately lead to the coalescence of the viral and host cell membranes and virus entry [10,11,7,8]. In the stable post-fusion six-helix bundle (6HB) arrangement (Fig 1C), which is believed to overcome the large free-energy barrier of membrane fusion [12], the C-HR helices pack in an antiparallel manner into conserved hydrophobic grooves on the surface of the central trimeric bundle of parallel N-HR helices [13,14,15,16,17].

No direct structural data are available yet that describe how the trimer switches from an extended prefusion state to the compact post-fusion 6HB. Indeed, because of their intrinsic dynamical and multi-state nature, the fusion intermediates are not easily accessible for studies using standard x-ray crystallography or cryo-EM techniques, and therefore their structural adaptations remain largely unknown [18]. Such studies also require conditions that mimic the optimal membrane environment, such as detergent micelles or bicelles, to maintain solubility particularly when the lipophilic FP, FPPR, MPER and TM regions are included in the constructs. The lipid-binding property of these proximal regions has been postulated to facilitate membrane fusion by destabilizing the viral and target cell membranes, thereby lowering the free-energy barrier for fusion [19,20,21]. In accordance, the emergence of a new class of membrane-conjugated peptide fusion inhibitors suggests direct interaction of the heptad regions with the cellular and/or viral membranes during the course of the fusion process [22,23,24].

Whether the fusion occurs exclusively at the cell surface or also within endocytic vesicles remains a matter of debate [25,26]. Several lines of evidence indicate that indeed analogues of gp41 consisting of the FP, N-HR, and C-HR exhibit pH-dependent fusion activity, consistent with an endocytic pathway [27,28,29].

In a recent solution NMR study, we described the structure of a construct which encompasses the N-HR and C-HR (Fig 1 and S1A Fig, residues 35–144) in dodecyl phosphocholine (DPC) micelles at pH 4 [30]. The truncations permitted a full resonance assignment and a complete structural analysis, showing that the trimeric 6HB conformation dissociates into monomers by interaction with DPC or T-cell mimicking phospholipid vesicles. Based on these results, we proposed a model of the fusion pathway where the C-HR and N-HR helices embed in the viral and host cell membranes, respectively, and directly participate in the fusion mechanism by providing the force pulling the two membranes into close juxtaposition. Such a force was proposed to counterbalance the lipid binding affinity of the ectodomain leading to the post-fusion 6HB conformation [30]. Other NMR studies encompassing the regions from FP to TM [31] and subsequently from FPPR to TM [32] at pH 4.0 in DPC showed that nearly all of FP and FPPR, most of N-HR, and IL regions give rise to relatively sharp NMR resonances but only very few of the CHR, MPER and TM resonances could be observed. Based on equilibrium sedimentation and small angle X-ray scattering analysis, both constructs were reported to be homotrimeric at the concentrations used in these NMR studies. The N-HR region was interpreted to be the principal determinant for trimerization with IL favoring this process [32], but the chemical shifts of the FPPR and N-HR were later shown to match very closely to the monomeric, lipid-bound state of these regions [33], suggesting that the TM is primarily responsible for maintaining the trimeric state of the protein at low pH.

The importance of the membrane proximal regions was emphasized in a crystallographic study of the trimeric gp41 ectodomain that included both FPPR and MPER segments. A 6HB arrangement was observed, revealing hydrophobic and limited hydrogen-bond interactions between the FPPR and MPER segments [17]. Although it is clear that these interactions will stabilize the post-fusion 6HB state, they can only form once the initially distant FPPR and MPER regions are brought in close proximity to one another. Whether such interactions initiate formation of the 6HB or stabilize a nascent 6HB cannot be established from the crystallographic data, but is important to develop a better understanding of the viral fusion mechanism. In a recent study, several gp41 constructs containing different combinations of the ectodomain and its membrane proximal segments, including FP and TM, but lacking the IL, were examined for their trimerization potential in DPC [34]. Longer constructs encompassing both FPPR and MPER showed trimer dissociation induced by low pH. Truncating the N-HR and C-HR regions in one such construct also abolished trimerization, even at pH 7, demonstrating that in the presence of phospholipid micelles, a delicate balance exists between the length of these helices and the equilibrium between monomer and 6HB assembly. Furthermore, these gp41 constructs were shown to induce leakage from liposomes, suggesting fusogenic activity, with higher lytic activity at pH 4 than at pH 7.

In the present study, we investigate the assembly of gp41 in the presence of detergent micelles by systematically examining the effect of construct length and pH on the monomer-trimer status of gp41. To this extent, we use a combination of sedimentation velocity (SV) measurements to determine oligomer status, and DEER EPR experiments to study trimer compactness by measuring distances at different positions of the 6HB. We use a favorable construct which exhibits good solubility in DPC and spans the regions from FPPR to MPER, while replacing IL with a 6-residue spacer [35] (S1C Fig, 17–172). We also find that low pH favors the dissociation in DPC while neutral pH shifts the equilibrium towards the trimer. Importantly, we show that interactions between the FPPR and a minimal region of MPER are required to maintain the trimeric arrangement at neutral pH. The trimeric 6HB formed by 17–172 resembles the structure of a shorter construct spanning just the N-HR and C-HR regions (34–144), but shows fraying of the C-terminal segment of MPER, likely a result of its association with DPC. Fraying at the N-terminal end of the FPPR appears much more restricted. MPER specific antibodies recognize the corresponding epitopes in the 17-172/DPC complex, exhibiting similar binding affinities to that of DPC-associated MPER peptide [36,37].

## Materials and Methods

### Recombinant gp41 constructs

DNA inserts, encoding amino acid sequences shown in S1 Fig, were synthesized and cloned in pJ414 vector (DNA 2.0, Newark, CA) or in pET11a (EMD Millipore, Billerica, MA) between 5’ Nde1 and 3’ BamH1 sites for expression in BL-21 (DE3) bacterial host. Expression and purification of constructs A, B and C through F (S1 Fig) have been described previously [30,33]. Construct B1 was purified similarly to that described for construct B. Construct G was derived from a variant of construct C bearing a 6His-tag at the N-terminus and the L6 spacer substituted with the sequence SGLVPRGSGG spanning a thrombin cleavage site. The final purified protein spanning gp41 residues 117–172 was attained by digestion with thrombin, followed by Ni-NTA affinity chromatography. The flow-through was subjected to reverse-phase high pressure liquid chromatography (RP-HPLC). Construct H was purified similarly to that described for construct C [33]. Construct I was expressed with a 6His-tag and a thrombin site at its N terminus (GSSHHHHHHSSGLVPRGS) and isolated similar to construct G. Except for construct D, the final step in the purification of all constructs used here involved RP-HPLC. Samples were dialyzed against 25 mM sodium formate, pH 3, at a concentration of 0.2–0.3 mg/ml, followed by exchange in 50 mM sodium acetate, pH 4. For constructs C through I, a final concentration of 10 mM DPC was added prior to dialysis. Samples were concentrated to 2–3 mg/ml and stored at 4°C until further use. This procedure showed the retention of ~1.5 to 2 equivalents of DPC micelle to one monomer or trimer. A schematic representation of the constructs is shown in S1 Fig and the sequences are listed in S2 Fig.

Variants of 17–172 (construct C) bearing substitution mutations R633C (17-172^R633C^) and L661C (17-172^L661C^), as well as a Cys residue introduced at the N- or C-terminus, termed ^N-Cys^17-172 and 17-172^C-Cys^ were also created (Fig 1), expressed and purified from inclusion bodies as described for construct C. A recombinant T20 peptide, GSGG-YTSLIHSLIEESQN QQEKNEQELLELDKWASLWNWF-C, spanning the Env sequence from Y638 to F673 and a C-terminal cysteine, was prepared and labeled with Alexa Fluor 647 similar to the protocol described previously for C34 tagged with the same dye [38].

### Sedimentation velocity (SV) experiments

Protein stock solutions maintained in 50 mM sodium acetate, pH 4, were diluted to a final concentration ranging from 10–20 μM in 20 mM sodium phosphate, pH 7, 150 mM NaCl or in 20 mM sodium phosphate, pH 6 (with or without 150 mM NaCl) or 50 mM sodium acetate at pH 4 and 5, all containing 10 mM DPC. They were dialyzed against the corresponding buffer containing 2 mM DPC for 5–6 hr, after which concentrations were measured based on their extinction coefficients at 280 nm prior to initiating SV experiments. pH dependent distribution of trimer to monomer was assessed using 20 μM protein in buffer containing 10 mM DPC prepared from the same stock solution of 17–172.

Sedimentation velocity experiments were conducted at 50,000 rpm and 20°C on a Beckman Coulter ProteomeLab XL-I analytical ultracentrifuge as described [33]. Samples were loaded in 2-channel centerpiece cells and scans were collected using both the absorbance (280 nm) and Rayleigh interference (655 nm) optical detection systems. Sedimentation data were time-corrected [39] and analyzed in SEDFIT 15.01b [40] in terms of a continuous c(*s*) distribution of Lamm equation solutions with a resolution of 0.05 S and a maximum entropy regularization confidence level of 0.68. Solution densities ρ and viscosities ƞ were calculated in SEDNTERP (http://sednterp.unh.edu/) [41]. Sedimentation coefficients *s* were corrected to *s*_*20*,*w*_ using partial specific volumes based on the complex protein:detergent stoichiometry as described [33]. The experimental protein:detergent stoichiometry was based on the integrated absorbance (protein alone) and interference (protein and detergent) signal for the major species observed in the *c(s)* distribution [42]. This stoichiometry was used to calculate the partial specific volume of the sedimenting protein-detergent complex and subsequently derive its molar mass [40].

In the case of samples prepared in a buffer containing 3.44 M glycerol, sedimentation velocity experiments were carried out at 60,000 rpm and 20°C. Absorbance sedimentation data were time-corrected [39] and analyzed in SEDFIT 15.01b [40] as described for the SV experiments without glycerol. To account for the co-sedimentation of glycerol and the accompanying formation of a dynamic density and viscosity gradient, an inhomogeneous solvent model was implemented [43]. The loading glycerol concentration was determined from a measurement of the refractive index of a control solution prepared under identical conditions in water. The sedimentation and diffusion coefficients of glycerol at 60,000 rpm and 20°C used in the analysis were determined experimentally by sedimentation velocity on dilute glycerol solutions. Density and viscosity data files describing the physical properties of glycerol solutions as a function of concentration were constructed using the coefficients in SEDNTERP [41], as described in http://www.analyticalultracentrifugation.com/inhomogeneous_solvent.htm. The zero order coefficients in the data files correspond to the density and viscosity of 50 mM sodium acetate. Excellent data fits were obtained with r.m.s.d. values of 0.0049 and 0.0051 A_280_.

### Circular dichroism

CD spectra were recorded in 20 mM sodium phosphate at pH 7 and 150 mM NaCl, 20 mM sodium phosphate at pH 6 (with or without 150 mM NaCl) and 50 mM sodium acetate at pH 4 and 5, all containing 10 mM DPC at 20°C on a JASCO J-810 spectropolarimeter using Spectra Manager software version 2 (Jasco Analytical Instruments, Easton, MD) and a 0.1 cm path length flat cell. Temperature melting experiments were carried out by recording the CD signal at 222 nm as a function of increasing and decreasing temperature. α-helical content was determined using the CDNN program [44].

### Isothermal titration calorimetry

Antigen 17–172 (construct C) and antibodies (Z13e1, 4E10 and 2F5 obtained from the NIH AIDS Research and Reference Reagent Program, Division of AIDS, NIAID, NIH) were diluted to a final concentration of ~60 μM (as monomer) and ~6 μM, respectively, and dialyzed against 10 mM Tris-HCl, pH 7.6, 150 mM NaCl, 2 mM DPC. Concentrations were estimated based on their 280 nm absorbance. Titrations were performed at 28°C on a iTC200 microcalorimeter (Malvern Instruments Inc., Westborough, MA). Results of control titrations of buffer with antigen were subtracted from the observed experimental results, and the data were processed using the Origin software provided with the instrument.

### DEER EPR

Fully deuterated (^2^H, ^12^C-d7) constructs 17-172^R633C^, 17-172^L661C^, ^N-Cys^17-172 and 17-172^C-Cys^ were expressed as described [33,45,38] and purified from inclusion bodies according to the protocol described for construct C [33], and verified by ESI-MS. An aliquot (~ 0.4 mg) of the protein following RP-HPLC and stored in ~35% acetonirile/water/0.05% TFA was dried down and labeled with deuterated MTSL (1-Oxyl-2,2,5,5-tetramethyl-Δ3-pyrroline-3-methyl)methanethiosulfonate, Toronto Research Chemicals, Toronto, Canada) as described [45,38]. Following fractionation of the labeled sample on a Superdex-75 column in 4 M guanidine HCl and 50 mM Tris-HCl at pH 7.5 to remove unreacted label, peak fractions were combined and concentrated. Samples were transferred from 4 M guanidine HCl, 50 mM Tris-HCl, pH 7.5, and 20 mM ^2^H DPC by dialysis against 20 mM sodium phosphate, pH 7, and 150 mM NaCl (EPR buffer). Concentrations were estimated based on A280 nm absorbance, and diluted to a final concentration of 50 μM in EPR buffer. A 50 μl aliquot was lyophilized and then dissolved to give ~50 μM final protein concentration in 70% D_2_O/30% deuterated glycerol (v/v) and ^2^H-DPC (≥ 2-fold excess of DPC micelles to protein monomer). When needed, additional ^2^H DPC was added from a stock solution of 0.5 M ^2^H-DPC dissolved in 99.9% D_2_O. T20 peptide was prepared as a 1.14 mM stock solution in 0.1 M sodium bicarbonate, pH 8, and added to 50 μl of a 50 μM solution of 17–172 to a final concentration of 1.2-fold molar excess over 17–172 (as monomer).

All four-pulse DEER data [46] were collected at Q-band (33.8 GHz) on a Bruker E-580 spectrometer equipped with a 150 W traveling wave tube amplifier and a model ER5107D2 resonator. All experiments employed 8-ns pump (ELDOR) π pulses, 12-ns π/2 and 24-ns π observe pulses, and a 95 MHz frequency difference between pump and observe pulses. The pump frequency was centered at the field spectrum maximum. The 400-ns half-echo periods of the first echo were incremented 8 times in 16-ns increments to average ^2^H modulation [47]. The pump pulse was incremented in 16-ns steps for ^N-Cys^17-172 and 17-172^C-Cys^ samples. All other experiments utilized 8-ns pump pulse increments. All samples were placed in 1.1-mm internal diameter quartz tubes (Wilmad WG-221T-RB) and flash frozen in liquid N_2_. All data were collected at 55 K. Total data collection times varied from 5 to 24 hrs. The window used for echo integration was 32–36 ns. The dipolar evolution data were truncated 700 ns before the end of the second echo period to avoid the “2+1” echo that results from excitation overlap of the pump and observe pulses. The P(r) curves were generated by Tikhonov regularization in DeerAnalysis2015 [48]. Ghost Suppression [49] for 3 spins was utilized in all fits. A dimension of 3.0 was used for all exponential background subtractions. The “Exci. bandwidth” function [50] of DEERAnalysis2015 was used with a bandwidth of 21 MHz to achieve a better representation of the short distance (< ~ 20 Å) region of the various P(*r*)’s.

### Size exclusion chromatography with multi-angle light scattering (SEC-MALS)

Molecular masses were estimated by analytical SEC with in-line MALS (DAWN Heleos-II, Wyatt Technology Inc., Santa Barbara, CA), refractive index (Optilab T-rEX, Wyatt Technology Inc.) and UV (Waters 2487, Waters Corporation, Milford, MA) detectors. Samples was applied to a pre-equilibrated Superdex-75 column (1.0 x 30 cm, GE Healthcare) and eluted at a flow rate of 0.5 ml/min in 10 mM Tris-HCl at pH 7, 150 mM NaCl and 2 mM DPC at room temperature. Molecular masses were calculated using the Astra software (version 6.1) provided with the instrument.

## Results and Discussion

### Designation of constructs and rationale

**Fig 2:**
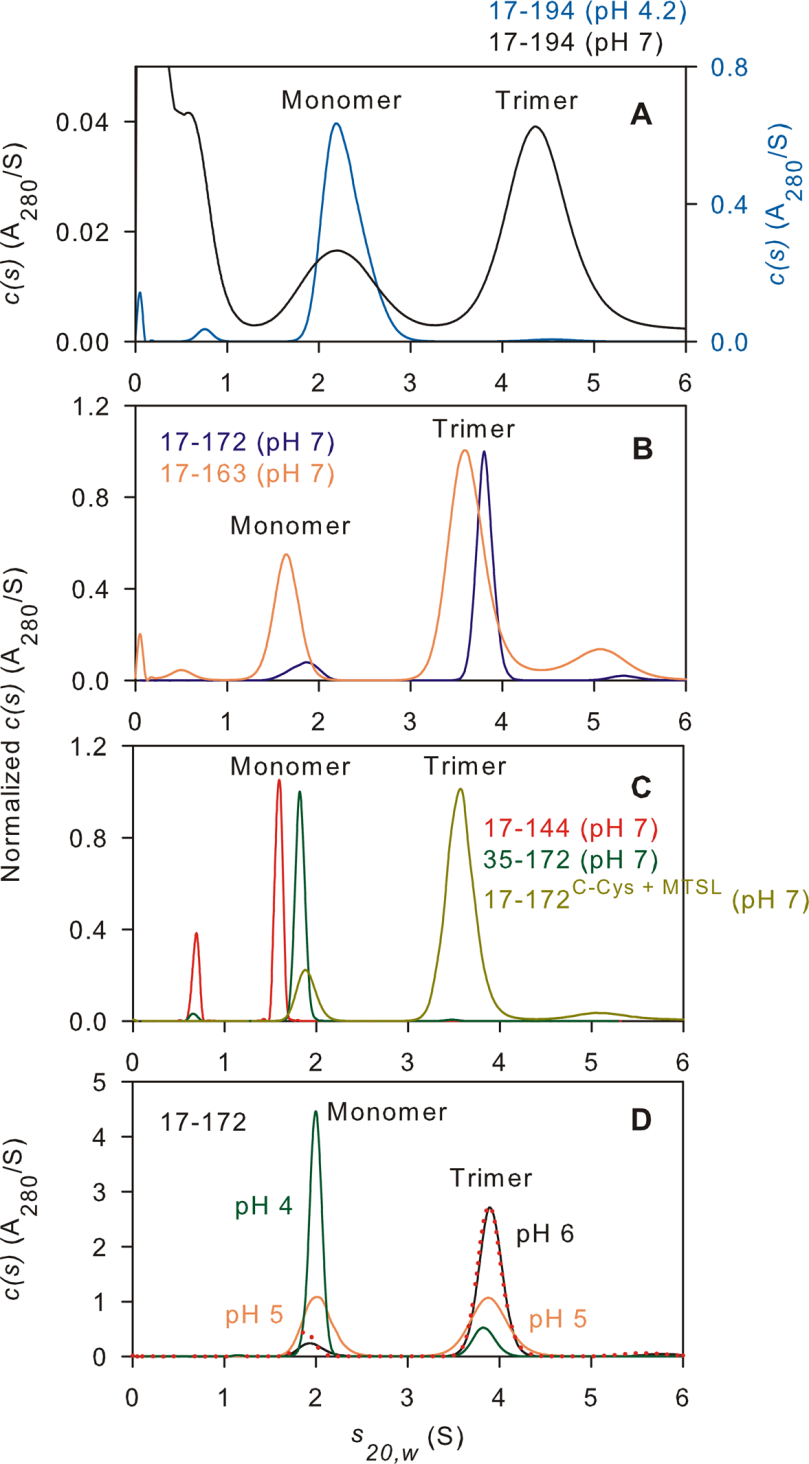
Sedimentation velocity absorbance *c(s)* distributions for various gp41 analogues at 20°C in the presence of an excess of DPC micelles. (A-C) Data collected in 20 mM sodium phosphate, pH 7 and 150 mM NaCl, except for 17–194 which was recorded in the absence of NaCl. Values derived for constructs A through I at pH 7 are listed numerically in Table 1. SV data for 17–194 at pH 4.2 was acquired in 50 mM sodium acetate with 25 mM KCl and 10 mM DPC (panel A, blue) and 17–172 at pH 4 and 5 (panel D: green and orange, respectively) in 50 mM sodium acetate with 10 mM DPC. Protein concentrations are as follows: 17–194 (pH 4.2), ~10 μM; 17–194 (pH 7), 1–2 μM; all others, 10–20 μM. The trimer/monomer distribution was unchanged in the absence or presence of 150 mM NaCl at pH 6 (panel D, red dotted trace). Slight variations in the sedimentation coefficients for the 17–172 and 17–172 ^Cys-MTSL^ trimer arise from differences in the isotopic labeling: ^2^H ^13^C ^15^N-labeled 17–172 complexed with ^1^H-DPC as compared to ^2^H-labeled 17-172^Cys-MTSL^ complexed with ^2^H-DPC.

Residues 512 to 705 of Env precursor are renumbered from 1–194 and named corresponding to the region they encompass (Fig 1, S1 and S2 Figs). In recent studies, we had shown that the core region (S1 Fig, construct B, 35–144) of gp41 comprising the N-HR and C-HR connected by a 6-residue spacer sequence (L6) forms a stable trimeric 6HB that is highly soluble across low (3.5 to 6) to neutral pH values (7.6) [51,38]. The trimer dissociation constant was shown to be below 250 nM at pH 7.6 in 10 mM Tris buffer containing 150 mM NaCl [38]. However, 35–144, as well as 35-144^IL^, the latter termed to denote the presence of the native IL sequence instead of the L6 spacer, are readily dissociated into monomers by DPC, both at below neutral pH (4–6) [30,33] and at pH 7 (Table 1). An extended construct (construct C, 17–172) which, unlike 35–144, requires a detergent environment for its solubility because it encompasses the FPPR and MPER regions, is also monomeric at acidic pH in the presence of ≥2-fold excess of DPC micelles as validated by sedimentation velocity experiments [33]. Performing experiments at pH 7 with gp41 constructs that span the entire ectodomain (see Fig 1) including the TM (1–194, [31,34]), or lacking just the N-terminal FP as in 17–194 (this work and reference [34]) is impractical due to their poor solubility. Indeed, shifting the pH of 17–194 from 4, where it is fully soluble in the presence of DPC, to 7 leads to significant aggregation. However, a small fraction of the protein that remains soluble at a low concentration (1–2 μM) showed two sedimenting species at ~2.2 and 4.4 S, corresponding to molar masses of 45 and 130 kDa, respectively. These 2 species represent detergent bound 17–194 monomer and trimer complexes (Fig 2A, black trace). This result is consistent with the observation by Dai et al. which showed that a construct encompassing the region 1–194, including the N-terminal FP and C-terminal TM, is trimeric, and is also poorly soluble at pH 7 in the presence of detergent [34]. However, construct 17–194 at pH 4.2 exhibits good solubility in the presence of DPC, evident from the nearly complete recovery of the absorbance signal, and is mainly monomeric (Fig 2A, blue trace)

**Table 1:** Mass estimations by sedimentation velocity analysis.

Construct	Concentration			Molar masses estimated by sedimentation velocity analysis		
	Protein	DPC	Protein:	*Mass*_experimental_ (kDa)	*Monomer mass*	*Major species*
	(μM)	(mM)	micelle ratio	[Sedimentation	(calculated Da)	
				coefficient (S)]		
*A)* 35-144^IL^	17	10–12	1: 8–9	32.3 (1.98)	16585*	Monomer + 45 DPC
*B)* 35–144	14.7	10–20	1: 9–18	24.4 (1.06)	8284	Monomer + 46 DPC
*B1)* 35–151	19.5	10	1: 7	24.9 (1.51)	10823*	Monomer + 31 DPC
*C)* 17–172	17	10–12	1: 8–9	67.0 (3.73)	14825*	Trimer + 64 DPC
*D)* 17–68 + 117–172	21.5	10	1: 6	25.1 (1.31)	17–68: 7425*	Monomer peptide + 25 DPC
					117–172: 8578*	
*E)* 17–144	21.5	10–12	1: 6–7	28.4 (1.60)	10782*	Monomer + 50 DPC
*F)* 35–172	18.4	10–12	1: 7–8	29.9 (1.85)	13678*	Monomer + 46 DPC
*G)* 117–172	17.5	10–12	1: 8–9	28.9 (1.39)	8204*	Monomer + 58 DPC
H) 17–163	20	10	1: 6.5	58.0 (3.64)	13460*	Trimer + 36 DPC
				17.6 (1.65)		Monomer + 37 DPC
*I)* 17-34(L6)147-163	20	10	1: 6.5	24.6 (0.96)	5065^@^	Monomer + 55 DPC
DPC		10		22.8 (0.52)	352	65 DPC

* and ^@^ denote ^2^H ^13^C ^15^N and H ^13^C ^15^N labeled samples, respectively.

As the construct 17–172, lacking the FP and TM regions, shows a much improved solubility at neutral pH in the presence of DPC, even at 100 μM concentration, it provided an ideal platform for further systematic studies by deletion mutagenesis, distance measurement between unique positions of the trimer, and molecular mass estimations in the presence of DPC, thereby offering insights into the elusive transitioning steps of the fusion process. We have made use of eleven gp41 analogues for the studies described here, as well as four constructs of 17–172 that each bear a single Cys substitution, needed for attaching the MTSL tags and for measuring distances when the trimer is in complex with the detergent micelle. Parallel SV analysis provided the stoichiometry of protein to detergent in the complex (see SV experiments in Materials and Methods).

### Domains encompassing FPPR to MPER are critical for stable 6HB formation in a detergent environment at pH 7

**Fig 3:**
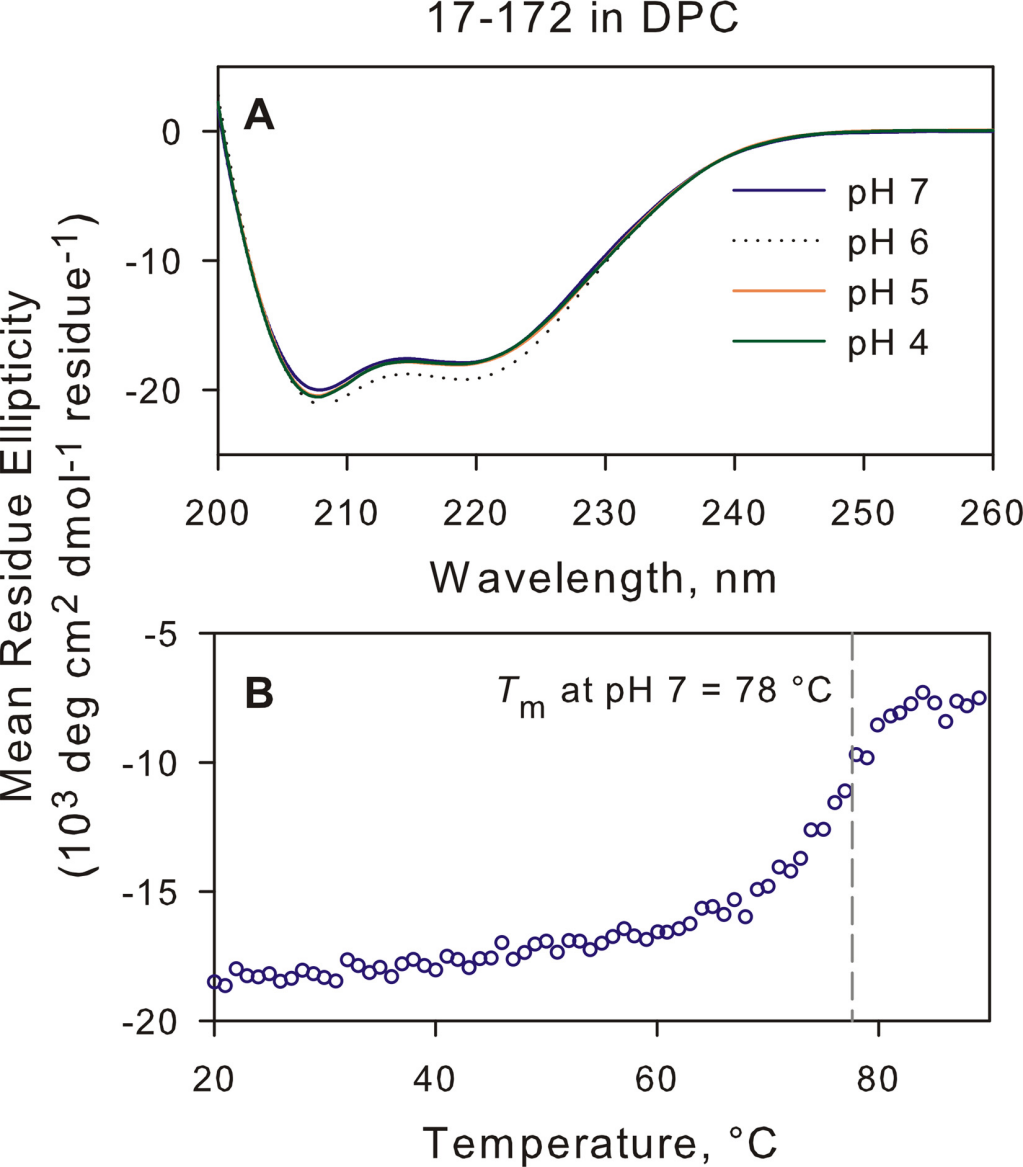
CD analysis of 17–172 in 10 mM DPC. (A) CD spectrum at 20°C of 17–172 at various pH values that were also subjected to SV analysis (see Table 1 and Fig 2). (B) Trace of the 222 nm CD signal of 17–172 trimer as a function of increasing temperature at pH 7.

The various gp41 analogues are shown in S1 and S2 Figs. All samples were prepared under nearly identical conditions in the presence of DPC at pH 7 (see Materials and Methods) and the results of the SV analysis are summarized in Table 1 and pertinent data are plotted for comparison. Constructs 35-144^IL^ and 35–144 (constructs A and B, respectively) are monomeric at pH 7, similar to results observed for these constructs at pH 4 [30,33]. Even a slightly longer construct B (B1, 35–151), extended by 3 residues flanking the C terminus of N-HR and the N terminus of C-HR, and with 7 residues at the C terminus of C-HR, also dissociates to a monomer in >1-fold excess of DPC micelles. However, construct 17–172 which encompasses the FPPR and MPER regions remains mainly a trimer upon addition of DPC (Fig 2B). It exhibits a helical content of ~ 64% (Fig 3A), close to the *ca* 70% value reported for the longer 1–194 construct [34]. The transition of 17–172 trimer to a monomer is clearly evident when monitoring the helical signature at 222 nm as a function of temperature, with a midpoint for this transition at ~78°C (Fig 3B). Because of the entropic advantage of having the N-HR and C-HR linked by the L6 spacer, the trimer stability of DPC-free 17–172 is expected to be higher than the *T*_m_ of 87.6°C shown for 17–172 when assembled as peptides in a 6HB arrangement in the absence of DPC [17]. A *T*_m_ decrease of 12°C was observed for the shorter 35–144 construct undergoing thermal transition either with (*T*_m_ = 80°C) or without (*T*_m_ = 68°C) the connecting spacer under identical neutral pH conditions in the absence of DPC [38]. These observations therefore suggest that although 17–172 forms a stable trimer at neutral pH, its thermal stability is significantly decreased upon binding to phospholipid micelles. The helical signature remains nearly identical at pH values ranging from 7 to 4 when transitioning from a trimer to a monomer with only a slight deviation at pH 6 (Fig 3A). Clearly, the dissociation of the 17–172 trimer at 20°C is evident below pH 6 (Fig 2D) with ~50:50 distribution of monomer to trimer at pH 5, similar to that reported for a construct encompassing the TM domain up to Env residue 711 [34]. A higher temperature of 36°C does not alter the trimer/monomer distribution at pH 6 or 7 (S3 Fig).

Deletion of MPER or FPPR as in constructs E (17–144) and F (35–172), respectively, leads to dissociation of the trimer (Fig 2C). Truncating the MPER at its C terminus by 9 residues starts to influence the monomer-trimer status as more monomer is observed for 17–163 (Fig 2B) relative to 17–172 under similar conditions. However, linking only the FPPR (17–34) and MPER (147–163) segments with a L6 spacer (construct I) does not lead to trimer formation indicating that N- and C-HRs together with FPPR and MPER are essential for trimer stability when bound to DPC. A similar construct design, spanning residues 1–44 and 138–194, and connected by a spacer was also shown to be mainly monomeric at pH 7 in DPC [34] suggesting that construct 35–144 likely represents the minimal N-HR and C-HR regions that together with FPPR and MPER are critical for trimer stability at pH 7 in DPC. Construct G (117–172) which spans just the C-HR and MPER regions is monomeric, a result expected for this polypeptide as it forms the outer helices of the 6HB when interfacing with the inner FPPR and N-HR helices.

By SV, we assess the average micelle size to be ~23 kDa or 65 DPC molecules and a S value of 0.52 under the conditions employed. We derived the approximate stoichiometry of trimer/monomer to DPC based on simultaneous monitoring of the absorbance at 280 nm and interference signal at 655 nm in our SV experiments. On average, the various constructs show association with ~1 DPC micelle, irrespective of a monomer or trimer. The 17–172 construct shows the most association, with a detergent mass that corresponds to *ca* 64 DPC molecules for the trimer.

### Trimeric conformation of 17–172 is recognized by MPER-specific antibodies

The antibody Z13e1 binds to an epitope between, and overlapping with, those of 2F5 and 4E10 (Fig 1) [36]. N671 and D674 within the minimal peptide epitope WASLWNWFDITN are crucial for peptide recognition and neutralization by Z13e1. However, the 17–172 construct used in our studies belongs to HXB2 isolate bearing D674N which may contribute to an altered affinity for binding of Z13e1 to 17–172.

**Fig 4:**
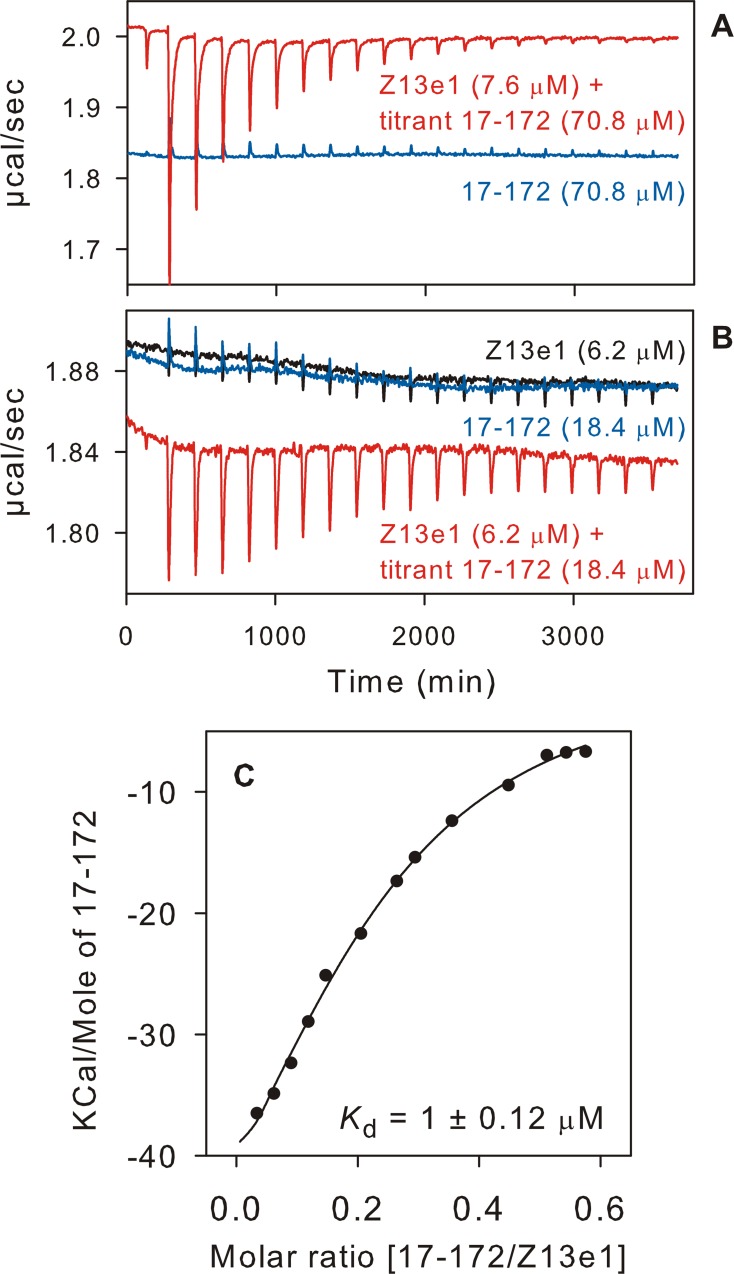
Binding isotherm for the interaction of Z13e1 with 17–172. The peaks in red indicate the heat released after each addition of 17–172 into the antibody solution. Traces in blue indicate control titrations using either just the antibody in the cell titrated with the buffer (10 mM Tris-HCl, pH 7.6, 150 mM NaCl, 2 mM DPC) shown in B (black) or the same buffer titrated with the antigen shown in A and B (blue). (C) The data were best fit using a single binding constant to calculate the thermodynamic parameters.

A ratio of 1:1.75 of 17–172 (as monomer, A280 nm) to DPC micelle (by NMR) was estimated for an unlabeled preparation of 17–172 trimer. As unmatched DPC concentration in the cell and titrant gave significant heat release, the antibody and the titrant (17–172) were dialyzed against 10 mM Tris-HCl, pH 7.6, 150 mM NaCl and 2 mM DPC for 5–6 hr prior to ITC. In an initial experiment, Z13e1 (7.6 μM) was titrated with an ~10-fold excess of 17–172. The resultant isotherm exhibited a majority of data points below the midpoint of the titration curve and an N value corresponding to <0.1 bound antibody (Fig 4A, red trace). This suggests that the amount of antibody competent to bind antigen 17–172 is significantly lowered, likely an effect of DPC. Titration with a smaller (~3-fold) excess of 17–172 gives a more sigmoidal isotherm (Fig 4B, red trace and 4C) with a somewhat higher apparent N-value of 0.22 ± 0.01 and an estimated *K*_d_ of 1 ± 0.12 μM at 28°C. This value is identical to the reported *K*_d_ for the binding of 4E10 to the lipid associated MPER peptide derived from the same HXB2 isolate [37], in spite of a reported 1-order of magnitude lower potency of Z13e1 relative to 4E10 against several pseudotyped HIV-1 [36].

For comparison with Z13e1, we also performed similar titrations of 4E10 and 2F5 with 17–172 (raw data shown in S4 Fig). Although accurate assessment was not possible because of the uncertain effects of DPC on the antibodies, the raw data (S4A and S4B Fig) indicate that epitopes to both antibodies exist even when the MPER region is presented as part of the 17–172 trimer complexed with DPC [52]. The fitted data yields a *K*_d_ of ~ 2.4 ± 0.3 μM for the binding of 2F5 to the MPER epitope in the 17–172 trimer (S4C Fig).

### Conformation of the terminal FPPR and MPER regions while in association with DPC micelles

**Fig 5:**
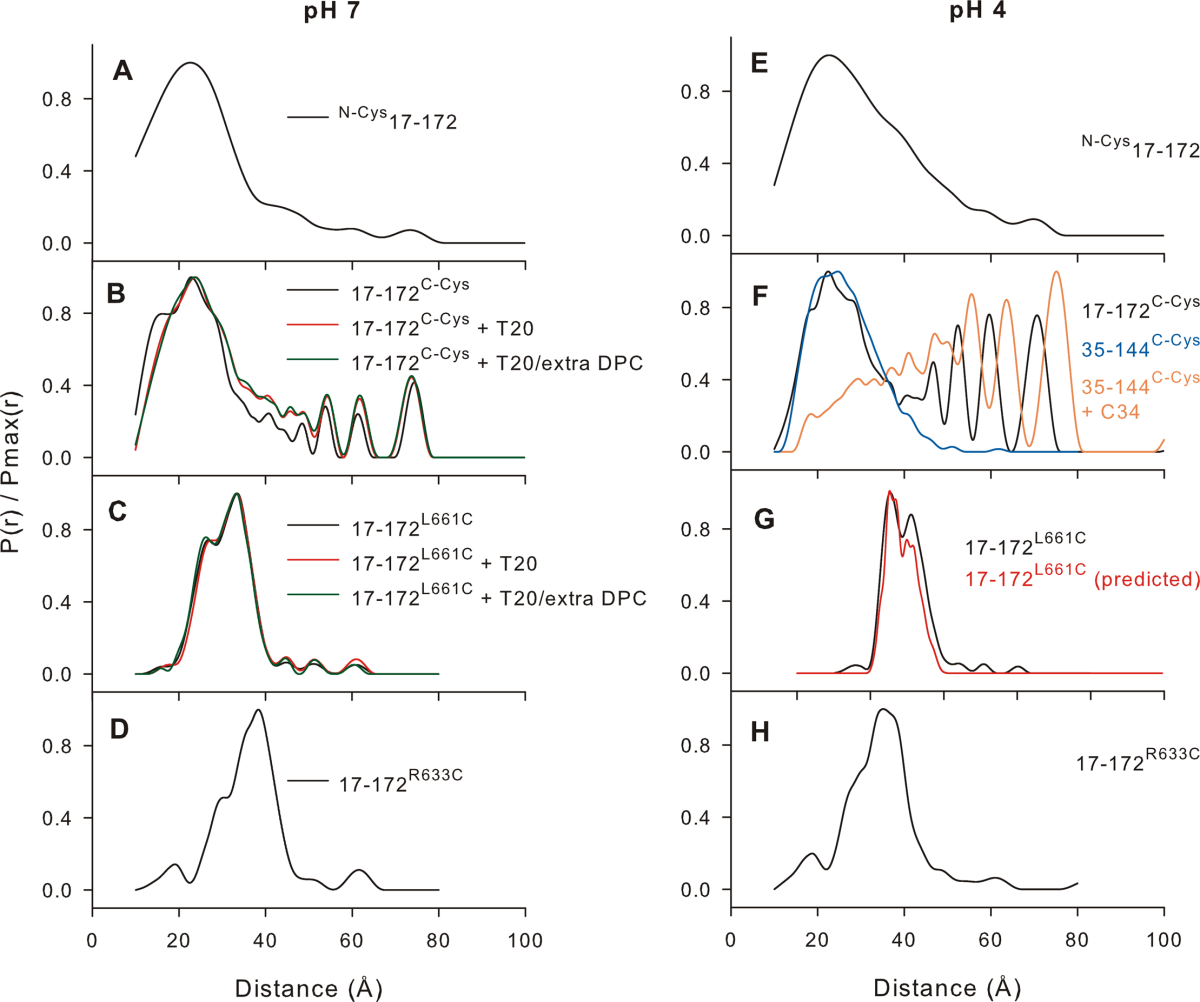
DEER EPR measurements with MTSL labels in various positions of the 17–172 construct. Positions of labels are shown in Fig 1. Results of the DeerAnalysis2015 Tikhonov Regularization fits [48] of the background corrected data acquired at pH 7 and pH 4 in the presence of ~2-fold excess of DPC micelles. The regularization parameter, α was determined by examination of the relevant L-curves (α = 10 in all cases except for the ^N-Cys^17-172 data where α = 1000 was the best choice). Plots of the raw data are shown in S6 and S7 Figs. Previously published data indicating the fraying of the C-HR region by the addition of C34 to the 6HB formed by the construct 35–144 (S1 Fig) in 10 mM Tris-HCL, pH 7.6, and 150 mM NaCl (without DPC) is superimposed (orange, panel F) for comparison with 17-172^C-Cys^ in the presence of DPC at pH 4. The blue trace depicts the distance distribution of 35–144 trimer in the absence of C34 and DPC at pH 7. P(*r*) distribution calculated using the MMM software package for 17-172^L661C^ labeled with MTSL is superimposed for comparison with the experimental data in panel G (red trace).

The 35–144 construct forms a stable trimer in the absence of DPC at pH 7.6 [38]. DEER [46] data acquired for 35–144 samples, with MTSL label attached either at the N- or the C-terminus, show narrow distributions of P(*r*) values consistent with a compact structure of the 6HB (Fig 5F, blue trace) [38]. However, in the presence of ~2-fold excess of DPC micelle to protein (as monomer) it is completely monomeric, as evident from SV data (Table 1) and DEER (S5 Fig). The dipolar evolution in the DEER experiment is essentially non-existent for 35–144 bearing a spin label at its C terminus (35-144^C-Cys^, S5C Fig). Similarly, for ^N-Cys^35-144, which bears a spin label at its N terminus, only the exponentially decaying inter-molecular background signal is observed with no apparent dipolar evolution. Thus, these data served as controls for exploring the compactness of DPC-associated 17–172 trimer through site-specific MTSL labeling and DEER measurements at pH 7 and pH 4.

Cysteine residues were introduced in the 17–172 construct as shown in Fig 1 and labeled with MTSL. The samples prepared for DEER measurements contain a ratio of ≥ 2 DPC micelles (~10 mM) per 17–172 monomer (50 μM). The pH 7 DEER results for 17–172 labeled at the C-terminus (17-172^C-Cys^, Fig 5B and 5F) reveal conformational heterogeneity. The width of the major population as well as the appearance of peaks at longer distances (> 40 Å) is consistent with structural heterogeneity that might arise from fraying or kinking of the MPER helix. Interestingly, the pH 4 data reveal a larger relative population of the MPER conformers with longer pair-wise MTSL distances and is, therefore, consistent with more fraying. The ^N-Cys^17-172 construct yields a P(*r*) distribution that is clearly broader than typical for a well-ordered protein structure (Fig 5A). Consistent with the 17-172^C-Cys^ DEER results, this distribution becomes wider as the pH is lowered from 7 to 4 (Fig 5B). It is possible that the fraying of the MPER affords less confined positioning of the helical FPPR and, hence, the slightly wider distribution. However, the possibility of helical fraying for a modest fraction of the trimer cannot be completely discounted by these results either.

**Fig 6:**
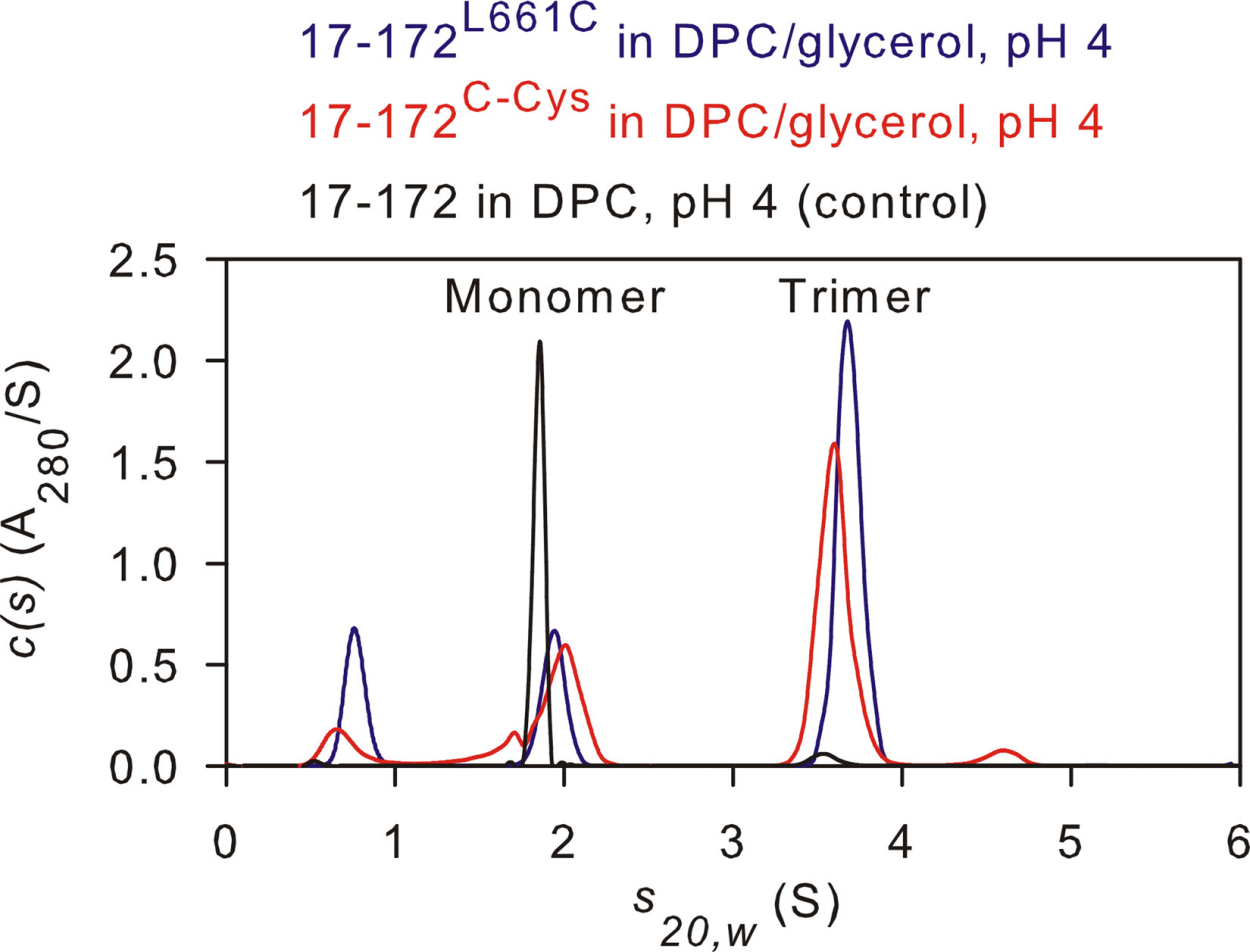
Sedimentation velocity absorbance *c(s)* distributions for MTSL-labeled 17–172. Experiments were carried out using 50 μM ^2^H-MTSL labeled, 17-172^L661C^ (blue) and 17-172^C-Cys^ (red) in 50 mM sodium acetate, pH 4, and 31% nondeuterated glycerol in the presence of ~2-fold excess of DPC micelles. Samples were prepared similar to those used for DEER at a concentration of 50 μM with the above buffer in H_2_O instead of D_2_O. 17–172 construct which is mainly a monomer in 50 mM sodium acetate at pH 4 and DPC, but in the absence of glycerol, is shown as control (black, rescaled to fit c(*s*) axis).

As noted above, lowering the pH from 7 to 4 resulted in a wider distribution of P(*r*) instead of a total loss in dipolar evolution, inconsistent with the SV data which showed that 17–172 completely dissociates into a monomer/DPC complex, as we reported previously at pH 4 and this work (black and green trace in Fig 6 and Fig 2D, respectively) [33]. This discrepancy can arise from 30% glycerol used in DEER measurements which alters the monomer-trimer equilibrium such that the 17–172 is destabilized but remains mainly a trimer, consistent with the larger P(*r*) distribution of the spin pair at the MPER C-termini. To evaluate this possibility, SV analysis were conducted with MTSL-labeled samples prepared in ~30% glycerol at pH 4. As shown in Fig 6, both 17-172^L661C^ and 17-172^C-Cys^ trimers each account for ≥70% of the total signal, indicating that glycerol shifts the monomer-trimer equilibrium towards the trimer. DEER measurements are sensitive to this shift in equilibrium as noted by the very broad P(*r*) distribution (compare Fig 5B and 5F). This observation is consistent with our published results [38] that displacement of the C-HR helix in the shorter 35–144 6HB trimer by the fusion inhibitor C34 also leads to a similarly broad distribution of P(*r*) values (Fig 5F, orange trace), contrasting with the narrow distribution observed for the same construct in the absence of C34 (Fig 5F, blue trace), both in the absence of DPC. These recently published results are superimposed strictly for comparison with 17-172^C-Cys^ DEER results in the presence of DPC [38]. The stabilization of the 17–172 trimer by glycerol is not uncommon and a similar stabilizing effect by glycerol was also observed for the human peripheral myelin protein 22, a membrane protein [53]. Thus, interpretations made from DEER measurements have to consider that conformational equilibria can be impacted by the high glycerol concentration.

P(*r*) distributions derived for samples labeled near the N- and C-termini of C-HR, namely R663C (Fig 1, 17-172^R633C^, Fig 5D and 5H), and L661C (Fig 1, 17-172^L661C^, Fig 5C and 5G), are typical of those for well-ordered proteins with labels attached to the surface. The widths of these P(*r*)’s are consistent with the range of MTSL rotamers calculated by the MMM software package [50] after the coordinate file of the construct 35–144 (PDB entry 1SZT, bearing a L661C mutation) was optimized by the SCWRL4 package [54] (Fig 5G, red trace). These results point to a compact, uniform structure for the C-HR region at pH 7. Shifting the pH from 7 to 4 likely alters the relative populations of preferred MTSL rotamers (especially for L661C), but there are no significant changes in the overall P(*r*) widths or distributions centers. Therefore, the distances between the three C-HR segments likely remains the same at these two pH values.

### T20 does not bind to the trimeric conformation of 17–172 in a DPC environment

T20, a first generation clinical fusion inhibitor T20 also known as Fuzeon and Enfuvirtide, is an N-acetylated linear 36-amino acid peptide that overlaps the C- and N-terminal residues of C-HR and MPER, respectively (residues 638 to 673 of Env, Fig 1) that is used in combination therapy for the treatment of HIV-1 infection [55]. It is predicted to bind to the exposed N-HR of the pre-hairpin state and perturb the assembly of the post-fusion 6HB arrangement. Thus, its interaction with 17–172 via possible displacement of the outer C-HR/MPER helices of the 6HB in a DPC environment can be easily judged by DEER. Addition of 1.2-fold molar excess of T20 to either 17-172^C-Cys^ or 17-172^L661C^ (as monomer) shows no significant changes in the overall P(*r*) widths or distribution centers (Fig 5B and 5C). Also, incubation of T20 labeled at its C-terminus with Alexa Fluor 647 dye showed no recovery of dye specific absorbance at 609 and 650 nm co-eluting with 17–172 trimer by size-exclusion chromatography in 10 mM Tris-HCl at pH 7.6, 150 mM NaCl and 2 mM DPC (data not shown). This result contrasts with recent experiments which showed C34 binding to the 35–144 trimer by displacement of the CHR in the same buffer without DPC [38]. These observations clearly indicate lack of any binding of T20 to the 17–172 trimer/DPC complex. Addition of an extra 10 mM final concentration of DPC to the same samples (Fig 5B and 5C) also yielded no significant changes in the overall P(*r*), demonstrating that the region spanning 17–172 forms a stable trimer embedded in DPC, consistent with the SV data under identical conditions at pH 7 (Table 1).

Lack of T20 binding to the 17–172 construct in excess DPC micelles is consistent with T20’s sequestration to the surface of DPC S8 Fig). This result also suggests that binding of T20 is likely to occur at the pre-hairpin state in a non-DPC-like environment rather than in the 6HB state, exemplified by 17-172/DPC complex. Also, under the same conditions where C34 binds to the 35–144 construct by displacing the C-HR in the absence of DPC, T20 exhibits no binding to 35–144 [38].

## Conclusions

The oligomeric arrangement of the gp41 ectodomain previously was shown to be a sensitive function of pH [27,28,29] and to be impacted by the presence of detergents and membranes [19,20,21]. For example, the 35–144 construct which effectively consists of the N-HR and C-HR helices, connected by a 6-residue linker, dissociates into monomers in the presence of either DPC or negatively charged phospholipid LM3 vesicles that mimic the T-cell membrane composition [30], but retains its 6HB arrangement in the presence of neutral SUVs composed of POPC. These results point to a delicate balance between the monomeric and trimeric states of the ectodomain, which is impacted by the presence of phospholipids, with negatively charged membranes and DPC favoring the dissociated state.

Using DPC as a reasonable mimetic for the interaction with actual membranes, thereby facilitating biophysical analysis, we have systematically analyzed the trimerization potential of the ectodomain of HIV-1 gp41, lacking the N-terminal FP and C-terminal TM (construct 17–172), by deletion mutagenesis, SV, and DEER measurements. Remodeling of the ectodomain while it transitions from the prefusion intermediate to the post-fusion 6HB state is key to the fusion process, and data on factors that modulate the oligomeric state of this domain are essential to gaining a better understanding of the fusion process.

The 35–144 construct, which forms the core of the gp41 ectodomain and includes the residues of N-HR and C-HR, either with the IL region or with the linker replaced with a 6-residue spacer (L6), clearly dissociates to a monomer in DPC at pH 7. We show that stable trimerization resembling the post-fusion 6HB conformation requires the presence of both the FPPR and the MPER regions flanking the N-HR and C-HR, respectively. Truncating the MPER region at its C terminus by 9 residues (construct 17–163) leads to a small increase in the monomer population relative to 17–172. However, linking just the FPPR and MPER regions with an L6 spacer yields only monomers in the presence of DPC. In contrast to what was seen previously at pH 4 for the longer 1–194 construct [31], that included the TM region, the 6HB conformation of 17–172 at pH 7 appears compact, with distances between spin-labels at the N- and C-termini of C-HR that are similar to those observed for the well-ordered 6HB core construct, 35–144, in the absence of DPC. Interestingly, distances measured by placing spin-labels at the N terminus of FPPR and C terminus of MPER indicate a heterogeneous distance distribution of the MPER relative to the FPPR. This may point to fraying of the terminal residues of MPER in the 17–172 construct, consistent with the kinking noted between residues W672 and F673 for the MPER peptide-DPC complex [37]. The region spanning W673-K683 then could represent the MPER portion that contacts the membrane and enables positioning of the flanking TM for a trimeric arrangement. Importantly, the Z13e1 antibody which spans the neutralizing 2F5 and 4E10 epitopes in MPER binds to the 17–172 trimer/DPC complex with an affinity of ~1 μM, similar to that of 4E10 binding to the MPER peptide/DPC complex [37]. In light of poorly resolved NMR spectra of constructs 17–172 and its analogues under various conditions, application of DEER provides a powerful alternative for the systematic analysis of the trimeric structure of lipid-associated full length gp41 ectodomain, and can contribute to gaining a better understanding of the intricacies of the fusion mechanism.

## Supporting Information

S1 Fig Various gp41 constructs used in this study and their designations. See Fig 1 for Env precursor numbering and S2 Fig for the exact sequence of each construct. Molar masses are listed in Table 1 and S2 Fig.
(PDF)

S2 Fig Amino acid sequences of gp41 constructs A through I listed in S1 Fig. Underlined sequences indicate nonnative residues.
(PDF)

S3 Fig Sedimentation velocity absorbance *c(s)* distributions for 17–172 at pH 6 and 7 at 36°C in the presence of excess of DPC micelles. Samples were prepared as described when carrying out the SV analysis at 20°C (see Fig 2D). For details, see Materials and Methods
(PDF)

S4 Fig Binding isotherm for the interaction of 4E10 or 2F5 with 17–172 at 28°C. (A and B) The peaks indicate the heat released after each addition of 17–172 into the antibody solution both maintained in 10 mM Tris-HCl, pH 7.6, 150 mM NaCl and 2 mM DPC. (C) The data for titration of 2F5 with 17–172 were best fit using a single binding constant to calculate the thermodynamic parameters.
(PDF)

S5 Fig Raw DEER data of fully deuterated 35–144 construct bearing a deuterated nitroxide-label. Labels were added either at the N (A and B) or the C terminus (C and D). DEER measurement was carried out in 10 mM Tris-HCl, pH 7.6, 150 mM NaCl either in the absence (B and D) or presence (A and C) of excess DPC micelles. Red traces are the exponential background functions employed to separate the random inter-molecular dipolar couplings from the desired intra-molecular dipolar couplings. The results of the DeerAnalysis2015 Tikhonov Regularization fit [48] of the background corrected data acquired in the absence of DPC is shown for 35-144^C-Cys^ (Fig 5F, blue trace). These previously published results [38] are shown here solely for the purpose of comparison with data acquired with the same constructs in the presence of DPC micelles (A and C).
(PDF)

S6 Fig Raw DEER data acquired with spin labels in different positions of the 17–172 construct at pH 7 in the presence of DPC. Red traces in all plots indicate the exponential background functions employed to separate the random inter-molecular dipolar couplings from the desired intra-molecular dipolar couplings. Panels A through D match with those shown in Fig 5 (left panels).
(PDF)

S7 Fig Raw DEER data acquired with spin labels in different positions of the 17–172 construct at pH 4 in the presence of DPC. Red traces in all plots indicate the exponential background functions employed to separate the random inter-molecular dipolar couplings from the desired intra-molecular dipolar couplings. Panel E through H match with those shown in Fig 5 (right panels).
(PDF)

S8 Fig Molecular mass estimation of T20 bound to DPC micelles by SEC-MALS. The plots show the T20-DPC micelle composition (B) as compared to an identical injection without T20 (A). The protein (black) and DPC-micelle (green) mass contributing to the combined mass (red) of the complex are indicated beside the peak. The RI trace (blue) matches with the trace of absorbance at 280 nm (black) consistent with the higher mass of one T20 bound to a micelle (B, ~30 kDa) by eluting earlier than the DPC-micelle peak (in A, ~26 kDa). The calculated mass of T20 peptide is 4492 Da.
(PDF)
