# Supplementary figures and images for: Insights into the Conformation of the Membrane Proximal Regions Critical to the Trimerization of the HIV-1 gp41 Ectodomain Bound to Dodecyl Phosphocholine Micelles
Source: PLoS One. 2016 Aug 11;11(8):e0160597. doi: 10.1371/journal.pone.0160597 (PMC4981318; doi:10.1371/journal.pone.0160597)

**S1 Fig. Various gp41 constructs used in this study and their designations.**

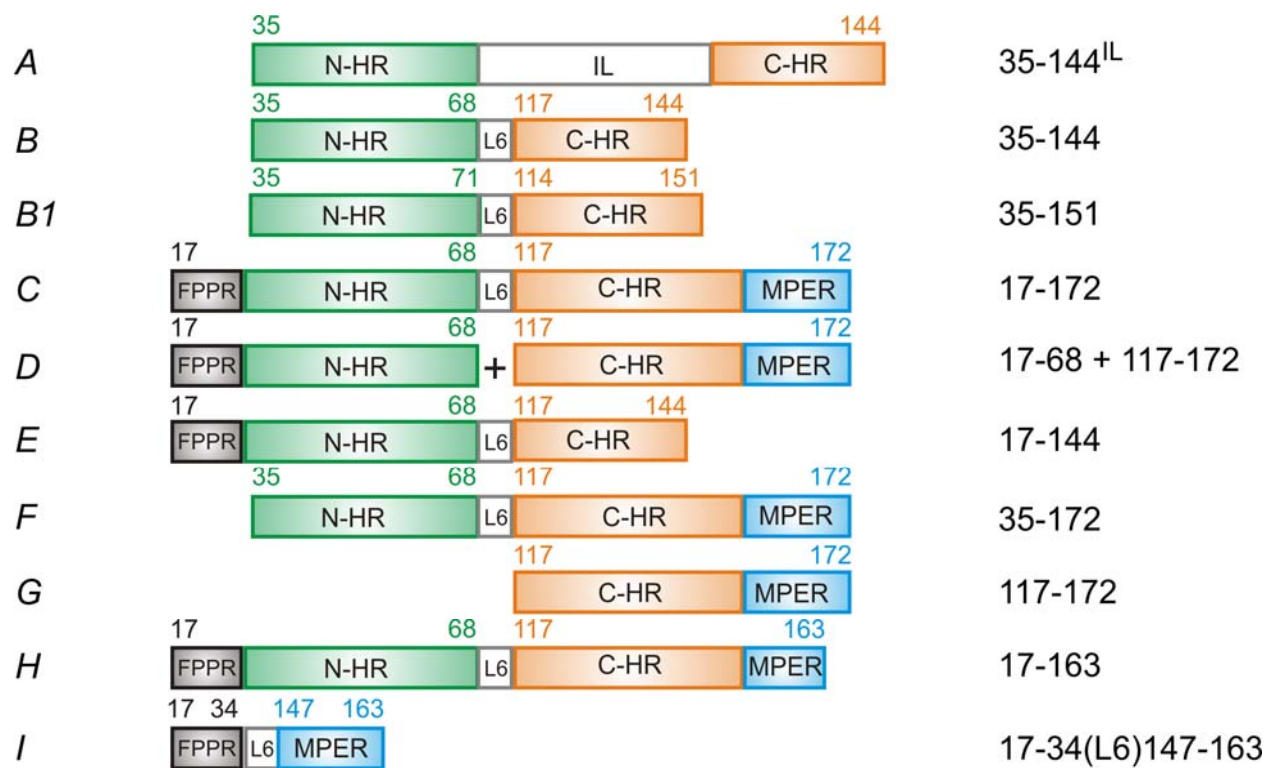

Supplement: S1 Fig — See Fig 1 for Env precursor numbering and S2 Fig for the exact sequence of each construct. Molar masses are listed in Table 1 and S2 Fig. (PDF) [file pone.0160597.s001.pdf]

**S4 Fig. Binding isotherm for the interaction of 4E10 or 2F5 with 17-172 at 28 °C.**

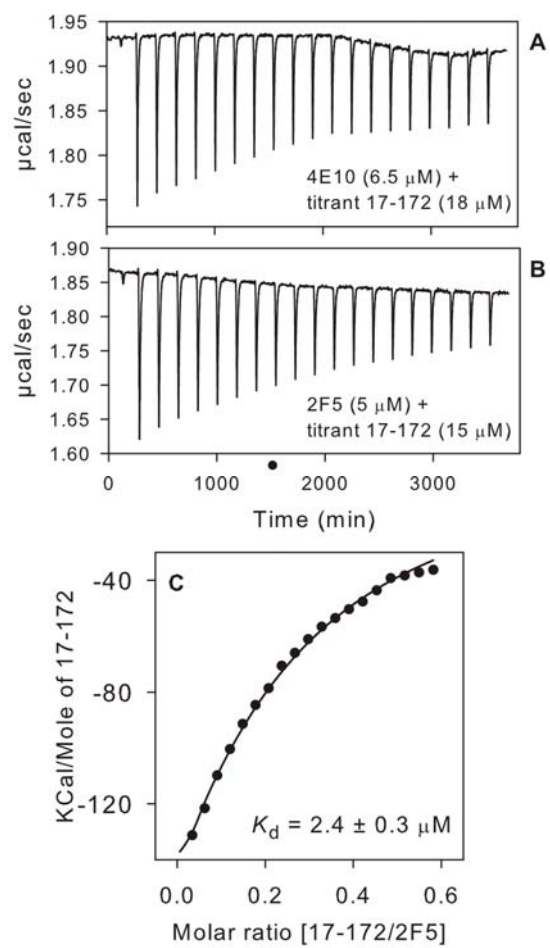

Supplement: S4 Fig — (A and B) The peaks indicate the heat released after each addition of 17–172 into the antibody solution both maintained in 10 mM Tris-HCl, pH 7.6, 150 mM NaCl and 2 mM DPC. (C) The data for titration of 2F5 with 17–172 were best fit using a single binding constant to calculate the thermodynamic parameters. (PDF) [file pone.0160597.s004.pdf]

**S5 Fig. Raw DEER data of fully deuterated 35-144 construct bearing a deuterated nitroxide-label.**

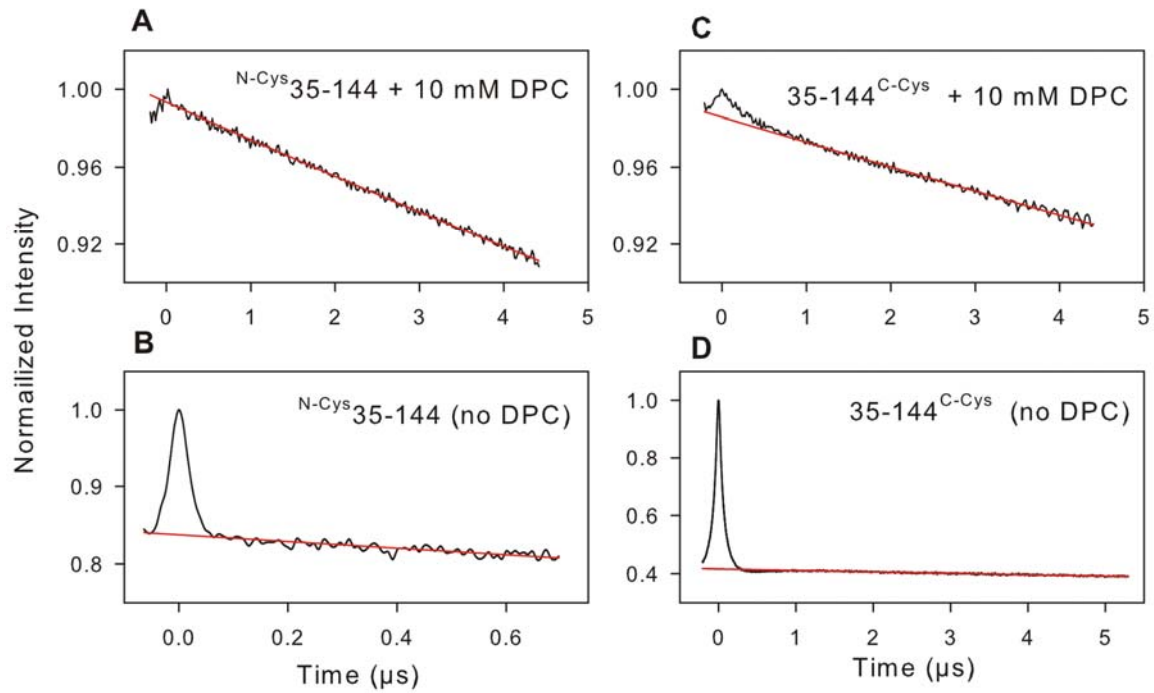

Supplement: S5 Fig — Labels were added either at the N (A and B) or the C terminus (C and D). DEER measurement was carried out in 10 mM Tris-HCl, pH 7.6, 150 mM NaCl either in the absence (B and D) or presence (A and C) of excess DPC micelles. Red traces are the exponential background functions employed to separate the random inter-molecular dipolar couplings from the desired intra-molecular dipolar couplings. The results of the DeerAnalysis2015 Tikhonov Regularization fit [48] of the background corrected data acquired in the absence of DPC is shown for 35-144C-Cys (Fig 5F, blue trace). These previously published results [38] are shown here solely for the purpose of comparison with data acquired with the same constructs in the presence of DPC micelles (A and C). (PDF) [file pone.0160597.s005.pdf]

**S8 Fig. Molecular mass estimation of T20 bound to DPC micelles by SEC-MALS.**

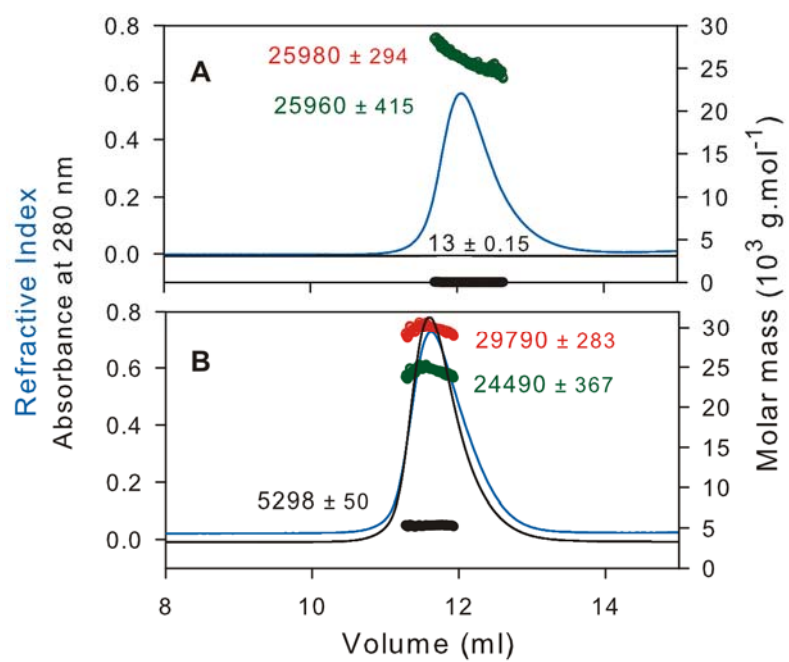

Supplement: S8 Fig — The plots show the T20-DPC micelle composition (B) as compared to an identical injection without T20 (A). The protein (black) and DPC-micelle (green) mass contributing to the combined mass (red) of the complex are indicated beside the peak. The RI trace (blue) matches with the trace of absorbance at 280 nm (black) consistent with the higher mass of one T20 bound to a micelle (B, ~30 kDa) by eluting earlier than the DPC-micelle peak (in A, ~26 kDa). The calculated mass of T20 peptide is 4492 Da. (PDF) [file pone.0160597.s008.pdf]
