# Supplementary material for: Insights into the Conformation of the Membrane Proximal Regions Critical to the Trimerization of the HIV-1 gp41 Ectodomain Bound to Dodecyl Phosphocholine Micelles
Source: PLoS One. 2016 Aug 11;11(8):e0160597. doi: 10.1371/journal.pone.0160597 (PMC4981318; doi:10.1371/journal.pone.0160597)
Supplement: S2 Fig — Underlined sequences indicate nonnative residues. (PDF) [file pone.0160597.s002.pdf]

S2 Fig. Amino acid sequences of gp41 constructs A through I listed in S1 Fig.

A) 35-144^{IL}

Expressed with a His-tag:

GSSHHHHHHS SGLVPRGSHM SGIVQQQNNL LRAIEAQQHL LQLTVWGIKQ LQARILAVER YLKDQQLLGI WGASGKLIAT TAVPWNASWS NKSLEQIWNH TTWMEWDREI NNYTSLIHSL IEESQNQQEK

Mass (^{2}H ^{13}C ^{15}N) – 16585.2

B) 35-144

Expressed with a His-tag:

GSSHHHHHHS SGLVPRGSHM SGIVQQQNNL LRAIEAQQHL LQLTVWGIKQ LQARSGGRGG WMEWDREINN YTSLIHSLIE ESQNQQEK

Final purified protein:

GSHMSGIVQQ QNNLLRAIEA QQHLLQLTVW GIKQLQARSG GRGGWMEWDR EINNYTSLIH SLIEESQNQQ EK

Mass (H ^{12}C ^{14}N) – 8283.9

B1) 35-151

Expressed with a His-tag:

GSSHHHHHHS SGLVPRGSHM SGIVQQQNNL LRAIEAQQHL LQLTVWGIKQ LQARILASGG RGGHTTWMEW DREINNYTSL IHSLIEESQN QQEKNEQELL E

Final purified protein:

GSHMSGIVQQ QNNLLRAIEA QQHLLQLTVW GIKQLQARIL ASGGRGGHTT WMEWDREINN YTSLIHSLIE ESQNQQEKNE QELLE

Mass (^{2}H ^{13}C ^{15}N) - 10822.9

C) 17-172

Expressed and purified from inclusion bodies:

STMGAASMTL TVQARQLLSG IVQQQNNLLR AIEAQQHLLQ LTVWGIKQLQ ARSGGRGGWM EWDREINNYT SLIHSLIEES QNQQEKNEQE LLELDKWASL WNWFNITNWL WYIK

Mass (^{2}H ^{13}C ^{15}N) – 14824.7

D) 17-68 + 117-172

Expressed and purified from inclusion bodies:

STMGAASMTL TVQARQLLSG IVQQQNNLLR AIEAQQHLLQ LTVWGIKQLQ ARILASGLVP RGSGGHTTWM EWDREINNYT SLIHSLIEES QNQQEKNEQE LLELDKWASL WNWFNITNWL WYIK

Final purified sequences for trimer assembly as peptides:

17-68:

STMGAASMTL TVQARQLLSG IVQQQNNLLR AIEAQQHLLQ LTVWGIKQLQ ARILASGLVP R

Mass (^{2}H ^{13}C ^{15}N) - 7425.4

117-172:

GSGGHTTWME WDREINNYTS LIHSLIEESQ NQQEKNEQEL LELDKWASLW NWFNITNWLW YIK

Mass (^{2}H ^{13}C ^{15}N) - 8577.6

E: 17-144

Expressed and purified from inclusion bodies:

STMGAASMTL TVQARQLLSG IVQQQNNLLR AIEAQQHLLQ LTVWGIKQLQ ARSGGRGGWM EWDREINNYT SLIHSLIEES QNQQEK

Mass (^{2}H ^{13}C ^{15}N) - 10782.1

F: 35-172

Expressed and purified from inclusion bodies:

SGIVQQQNNL LRAIEAQQHL LQLTVWGIKQ LQARSGGRGG WMEWDREINN YTSLIHSLIE ESQNQQEKNE QELLELDKWA SLWNWFNITN WLWYIKGSGK KKKD

Mass (^{2}H ^{13}C ^{15}N) - 13677.5

G: 117-172

Expressed with a His-tag:

GSSHHHHHHS SGSTMGAASM TLTVQARQLL SGIVQQQNNL LRAIEAQQHL LQLTVWGIKQ LQARSGLVPR GSGGWMEWDR EINNYTSLIH SLIEESQNQQ EKNEQELLEL DKWASLWNWF NITNWLWYIK

Final purified protein:

GSGGWMEWDR EINNYTSLIH SLIEESQNQQ EKNEQELLEL DKWASLWNWF NITNWLWYIK

Mass (^{2}H ^{13}C ^{15}N) - 8204.3

H) 17-163

Expressed and purified from inclusion bodies:

STMGAASMTL TVQARQLLSG IVQQQNNLLR AIEAQQHLLQ LTVWGIKQLQ ARSGGRGGWM EWDREINNYT
SLIHSLIEES QNQQEKNEQE LLELDKWASL WNWFN

Mass (^{2}H ^{13}C ^{15}N) - 13460.4

I) 17-34(L6)147-163

Expressed with a His-tag:

GSSHHHHHHS SGLVPRGSST MGAASMTLTV QARQLLSGGR GGEQELLELD KWASLWNWFN

Final purified protein:

GSSTMGAASM TLTVQARQLL SGGRGGEQEL LELDKWASLW NWFN

Mass (H ^{13}C ^{15}N) - 5065.4
