# Supplementary material for: Insights into the Conformation of the Membrane Proximal Regions Critical to the Trimerization of the HIV-1 gp41 Ectodomain Bound to Dodecyl Phosphocholine Micelles
Source: PLoS One. 2016 Aug 11;11(8):e0160597. doi: 10.1371/journal.pone.0160597 (PMC4981318; doi:10.1371/journal.pone.0160597)

S3 Fig. Sedimentation velocity absorbance *c(s)* distributions for 17-172 at pH 6 and 7 at 36 ºC in the presence of excess of DPC micelles.

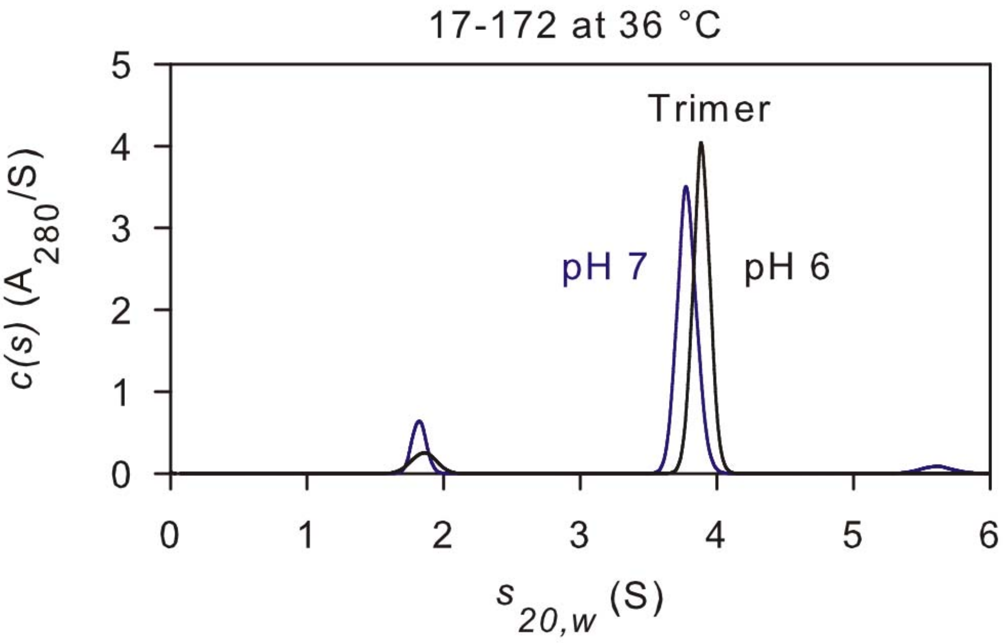

Supplement: S3 Fig — Samples were prepared as described when carrying out the SV analysis at 20°C (see Fig 2D). For details, see Materials and Methods (PDF) [file pone.0160597.s003.pdf]
