# Supplementary material for: Insights into the Conformation of the Membrane Proximal Regions Critical to the Trimerization of the HIV-1 gp41 Ectodomain Bound to Dodecyl Phosphocholine Micelles
Source: PLoS One. 2016 Aug 11;11(8):e0160597. doi: 10.1371/journal.pone.0160597 (PMC4981318; doi:10.1371/journal.pone.0160597)

S7 Fig. Raw DEER data acquired with spin labels in different positions of the 17-172 construct at pH 4 in the presence of DPC.

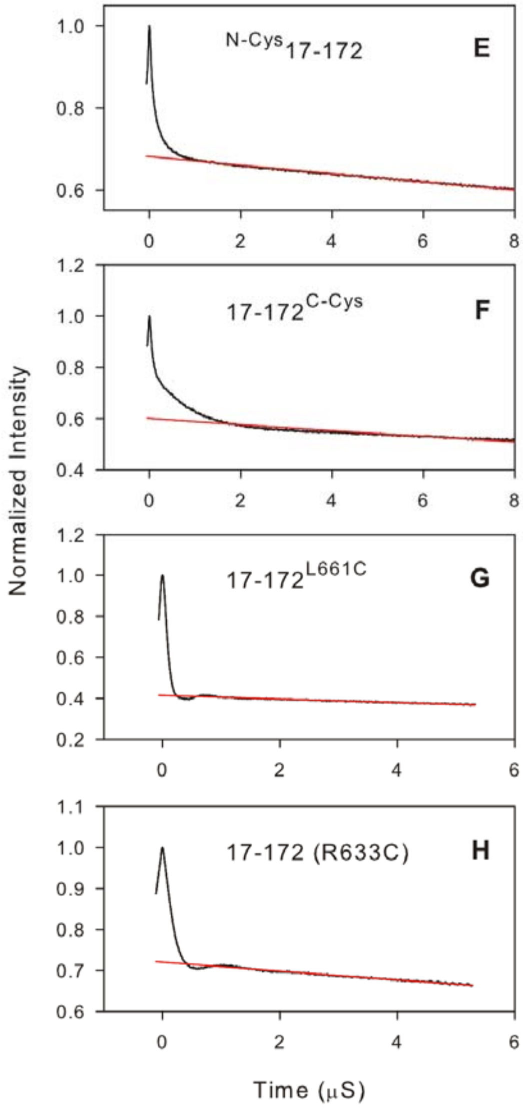

Supplement: S7 Fig — Red traces in all plots indicate the exponential background functions employed to separate the random inter-molecular dipolar couplings from the desired intra-molecular dipolar couplings. Panel E through H match with those shown in Fig 5 (right panels). (PDF) [file pone.0160597.s007.pdf]
